# Post-infection physeal bar resection of the proximal tibia: a case report and narrative review of the literature

**DOI:** 10.3389/fped.2026.1866624

**Published:** 2026-07-17

**Authors:** Roman Shcherbakov, Ahmer A. Khan, Ardian Ramadani, Andreas Tsoupras, Giacomo de Marco, Oscar Vazquez, Christina Steiger, Romain Dayer, Nicolas Lutz, Dimitri Ceroni

**Affiliations:** 1 Faculty of Medicine, University of Lausanne, Lausanne, Switzerland; 2 Pediatric Orthopedics and Traumatology Unit, Geneva University Hospitals and University of Geneva, Geneva, Switzerland; 3 Paediatric Orthopaedics Department, Children’s Hospital, Lausanne, Switzerland

**Keywords:** arrest, bar, growth, paediatric, physeal, post-infection, resection

## Abstract

The formation of physeal bars following growth plate injuries can cause complications such as limb length discrepancies and angular or physeal deformities. Physeal bars usually form after fractures but can also develop due to infectious processes. Management strategies depend on factors such as the bar's location, extent and residual growth potential. We describe the case of a two-year-old female with a lower limb discrepancy and a slight varus knee deformity caused by a proximal tibial physeal bar due to a neonatal infection. The severe anatomical epiphyseal anomaly of her right proximal tibia had developed into a consistent joint irregularity. At 26 months old, the patient underwent a physeal bar resection, assisted by arthroscopy and intraoperative C-arm imaging. This minimally invasive technique facilitated safe, accurate removal of the lesion, avoiding any resection of healthy physeal cartilage. Postoperative outcomes were favourable, including restored range of knee motion and full symptom resolution. This approach demonstrated the clinical value of integrating arthroscopy equipment assistance with intraoperative C-arm imaging during the surgical treatment of physeal bars.

## 1 Introduction

Premature growth arrest is a phenomenon characterised by an unexpected discontinuation of longitudinal and/or appositional bone growth secondary to an injury to the growth plate before skeletal maturity (1). Growth usually ceases due to a brake on growth called a physeal bar—an area of physeal growth arrest due to the formation of bony or fibrous bridges between the epiphysis and metaphysis (2). These bars act as a tether to normal longitudinal bone growth, which can then cause limb length discrepancies (LLDs), angular deformities, articular abnormalities or all three simultaneously (3, 4).

Physeal bars usually result from trauma, but they may also be the sequelae of sepsis, transphyseal osteomyelitis, neoplasia, vascular insult, thermal injury, radiotherapy, environmental exposure, congenital deformities or even medical treatment (5, 6). From a pathophysiological standpoint, clinicians may encounter several different bar formation mechanisms. One is thought to occur after a local restriction of the physeal blood supply following a variety of possible physeal injuries, resulting in abnormal local bone deposition. Physeal bars may also form due to a fracture's malunion following trauma, leading to an abnormal fusion between epiphyseal and metaphyseal bone (7, 8).

Physeal bars may be peripheral, central or linear, with their location and extent determining the effects on the subsequent growth of the incriminated physis (9). Once a physeal bar develops, that specific part of the physis will no longer contribute to the bone's linear growth. Thus, a large, centrally positioned bar in a child with significant growth remaining will result in a considerably shorter limb. Peripheral bars can lead to potentially severe angular deformities, which compromise joint mechanics and limb alignment.

A bar's location and extent are currently determined by using either computed tomography (CT) or magnetic resonance imaging (MRI). CT is particularly helpful for the three-dimensional characterisation of physeal bars, especially for preoperative planning (2). Fibrous physeal bars can be seen as subtly denser regions of soft-tissue attenuation relative to the lower-density cartilage, but they are easily missed and poorly distinguishable on radiographs and can even be missed using CT (10, 11). Thus, MRI seems to constitute the most appropriate method for detecting early fibrous bars, while also providing precise information on established osseous bars (12, 13).

The management of physeal bars aims to prevent any progression of deformity and preserve normal limb alignment and growth whenever possible. Treatment options range from observation in mild cases to surgical interventions such as physeal bar resection, epiphysiodesis, hemiepiphysiodesis, corrective osteotomy or even bone lengthening. Thus, the treatment choice must be tailored to the individual patient, but above all to the bar's location, extent and aetiology, and with an eye to the remaining physeal growth, to ensure the optimum outcome.

We report on a 26-month-old female who presented with significant physeal arrest of the right proximal tibia following a neonatal infection. Surgical treatment involved a complete resection of the physeal bar, followed by the interposition of nonresorbable bone wax. This study provides a glimpse into the domain of surgical bar resection by highlighting a case with a large surface area that would previously have been thought irreversible.

## 2 Case report

A 24-month-old female was referred to our paediatric orthopaedics unit for a lower-limb length discrepancy and varus knee deformity that had progressed over time. There was no history of trauma, physical abuse or fever, and the parents explained that their child had sustained a neonatal infection that had been treated at a hospital abroad. Although they possessed no documents to corroborate their statements, they clearly remembered that their child had to be admitted to an intensive care unit due to septic shock. Over the next two years, the parents noted a disparity in the length of their child's lower limbs, which progressed slowly but inexorably. This was accompanied by a varus deformity centred on the knee. These morphological and static disorders of the limb were not accompanied by any functional impairment of the knee. Even though the infectious origin of the transphyseal bar is the most probable, the diagnosis remains presumptive rather than definitively confirmed.

**Figure 1:**
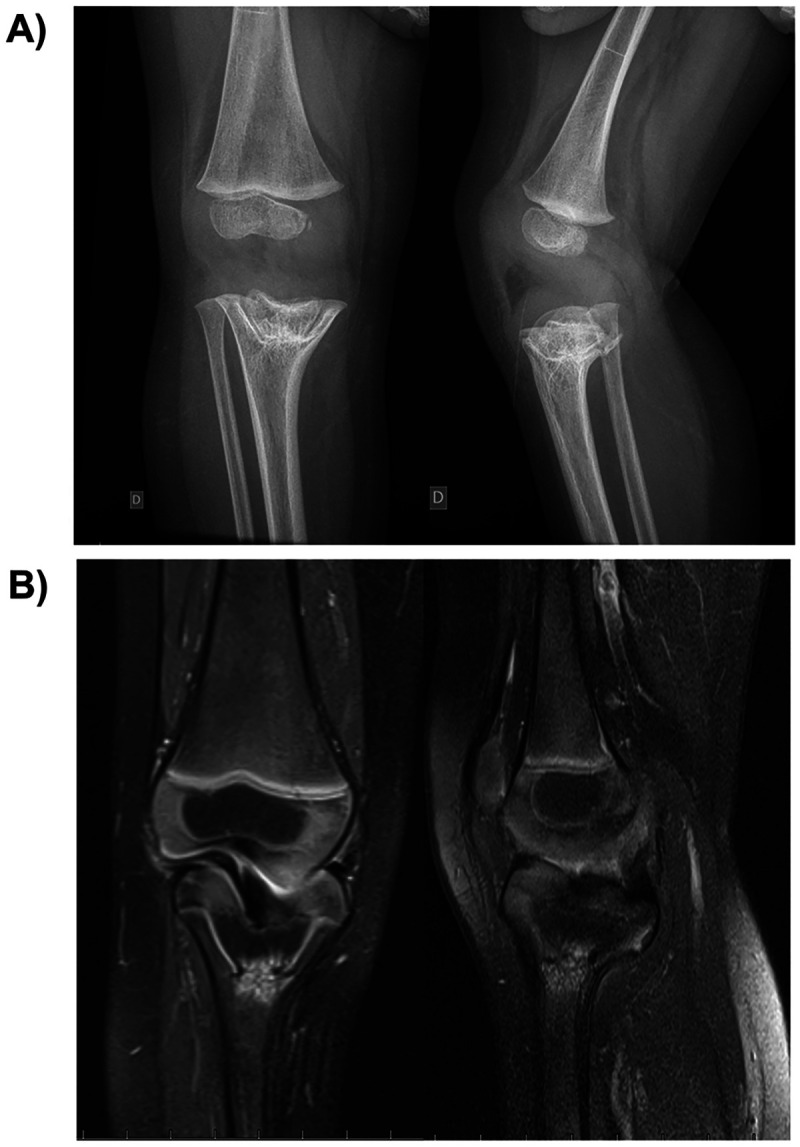
**(A)** primary anteroposterior and lateral radiographs of the right knee showing severe damage to the proximal tibial physis, with a depression of the epiphysis into the metaphysis, suggestive of a central physeal bar involving approximately 33% of the physeal surface. **(B)** MRI of the right knee showing a markedly concave deformity of the proximal tibial epiphysis, with a central fossa and cupping, associated with significant remodelling of the adjacent medial femoral condyle.

**Figure 2:**
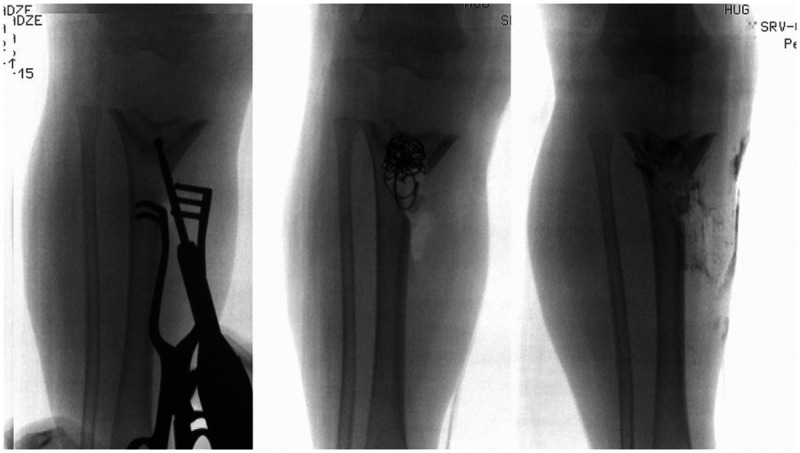
Intraoperative fluoroscopic views illustrating resection of the central proximal tibial physeal bar through an ascending metaphyseal tunnel under fluoroscopic guidance, with subsequent filling of the cavity using no resorbable bone wax.

**Figure 3:**
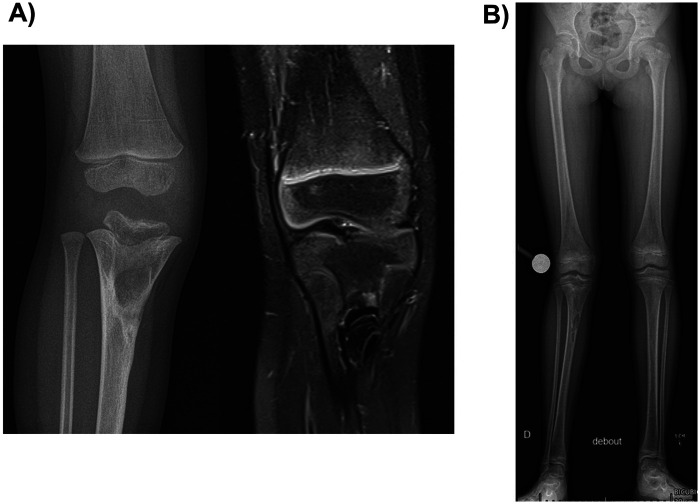
**(A)** follow-up radiograph and MRI demonstrating progressive restoration of the proximal tibial epiphysis toward its normal position, with normalisation of its shape and resolution of the cupping deformity. **(B)** Standing long-leg radiograph obtained 10 years after surgery showing tibial overgrowth associated with a mild valgus deviation of the right lower limb.

An initial radiographic evaluation, based on plain anteroposterior and lateral x-ray images, demonstrated severe damage to the tibia's proximal epiphysis, which appeared to have been ’sucked into’ the metaphysis due to the formation of a central bar estimated to cover 33% of the physeal surface (Figure 1A). A leg length discrepancy was present, measuring 0.7 cm. At the same time, there was a varus of the proximal tibia estimated at 10° (mechanical proximal tibial medial angle 77°). MRI revealed that the proximal epiphysis had taken on a pronounced concave shape, with a central fossa and cupping that had produced significant changes in the adjoining medial condyle (Figure 1B). Since the central physeal bar predisposed the child to future severe anatomical epiphyseal anomalies of the proximal tibia, which could lead to irremediable joint irregularities, clinicians recommended that the parents’ consent to a resection of the central bar. Surgery was planned for two months later, and the bar resection was performed using a burr under radioscopic control and arthroscope assistance through an ascending metaphyseal canal (Figure 2). Using the arthroscope to assist in central bar removal improved the visualization of the healthy peripheral physis, and thus limits the excessive resection of healthy growth cartilage. The contribution of the C arm during the procedure was also important to avoid excessive bone resection, particularly in the subchondral bone. The cavity was then filled with non-resorbable bone wax to prevent the reemergence of a physeal bar. Two months after the intervention, the beginning of proximal migration of the physis could be observed on the radiographs. In fact, we noted during follow-up a progressive migration of the epiphysis into its natural position, with a normalisation of its shape and the resolution of the cupping deformity (Figure 3A). Ten years after surgery, we observed some hypergrowth of the tibia (17 mm) with only a slight valgus deviation (8°) (Figure 3B).

## 3 Literature search strategy

Our narrative review of the literature was based on a critical reading of articles tracing the onset of physeal bars and the surgical experience acquired during their resection. We conducted a comprehensive search of relevant databases, including PubMed, Scopus, MEDLINE, Embase, CINAHL and Google Scholar, for English-language publications reporting original research and published before December 2025. To aid in the screening process, we used the keywords paediatric, growth, arrest, physeal and bar, defined Boolean combinations following the recommendations of Pai *et al*. (14) and had the assistance of an experienced research librarian. This strategy was supplemented by a manual search of the references in each retrieved article, thus identifying further potentially interesting works. Two authors (RS and DC), blinded to each other, separately screened all the search results based on our inclusion and exclusion criteria.

The review included studies involving paediatric patients under 16 years old with confirmed growth arrest due to radiologically confirmed physeal bars. Original articles were included if they were published in a peer-reviewed journal or available in a database, and the full text was available. Observational and randomised controlled trials were included in this narrative review, as were well-documented case series including at least five patients. Case reports, case series of four patients or fewer, review articles, technical reports, letters to the editor, experimental animal studies and published abstracts were excluded.

The review analysed demographical data (sexe, age,…), epidemiological data of physeal bars (aetiology, anatomical site, …), preoperative radiological analyses of physeal bars (location, size, …), the surgical strategies adopted (age at surgery, surgical procedure, …), and the postoperative and follow-up outcome measures to assess the effectiveness and safety of treatment approaches involving bar resection. Combining information on each of these steps would thus provide greater insight into the overall safety, efficiency and success of this treatment method. We used purely descriptive statistics, including means, medians, and ranges, to organize and interpret the available data in a transparent manner in the two tables available in the manuscript.

Our literature search identified 144 publications, but after reading their abstracts, 129 were excluded either because of an inappropriate study design or publication type, the wrong population type or a focus on experimental animal studies. The 15 remaining studies examined 271 patients and formed the bibliographic foundation for our reflection.

## 4 Discussion

Delayed diagnosis or treatment of osteoarticular infections in children can lead to substantial morbidity and serious complications. Infection may impair growth through several mechanisms, with or without direct involvement of the adjacent bone, and each can ultimately result in physeal bar formation. Thus, the physis may be affected by infection through multiple pathways (15).

Bacteria and host inflammatory cells usually reach the physis after substantial metaphyseal involvement. Bacterial enzymes, toxins and the inflammatory response may damage local tissues, leading to bone necrosis and sequestrum formation. Severe or prolonged osteomyelitis can therefore directly injure the growth plate, causing focal physeal lesions or transphyseal bone abscesses. Growth impairment may also occur without adjacent bone infection. In these cases, septic shock, disseminated intravascular coagulation or infectious vasculitis may compromise the physeal blood supply through ischaemic changes (15, 16). Thus, systemic effects, rather than local infection itself, may account for the growth disturbance.

Septic shock, characterised by persistent hypotension and severe perfusion abnormalities despite adequate fluid resuscitation, can compromise peripheral or non-vital tissues, including bone, particularly when inotropic or vasopressor support is needed. Disseminated intravascular coagulation may further impair tissue perfusion by causing widespread microthrombi that obstruct small vessels and disrupt organ function. The physis is especially susceptible because of its high metabolic demand, specialised vascular anatomy and rich blood supply. Prolonged ischaemia may damage or destroy the reserve zone of the physis (17), resulting in germinal chondrocyte necrosis. In neonatal infections, physeal injury is therefore often predominantly ischaemic, which may explain both the size of the lesion and later physeal bar formation.

This case report highlights the importance of classifying physeal bars by timing, location, extent and healing pattern, as these features determine residual growth and functional prognosis. Physeal bars may be peripheral, central with an intact periphery, linear or extensive (16, 18). Peripheral bars typically develop soon after infection and worsen rapidly with growth, producing angular deformities in any plane according to their position. Central bars, located nearer the midline, usually cause less angular deviation, but their onset is less predictable: they may appear early or, as in this case, become apparent only about two years after infection. Around the knee, central bars can lead to marked limb-length discrepancy despite limited angular deformity. Articular changes, including tenting, cupping, forking or flattening, are also common, particularly after neonatal infection (16, 18).

The conventional indications for physeal bar resection—less than 50% involvement of the growth plate and at least two years of remaining growth—have remained largely unchanged (19, 20). Resection is generally recommended when less than half of the physis is affected, more than two years of growth remain, and the predicted limb-length discrepancy is greater than 2 cm (20, 21). Langenskiöld, who pioneered this procedure, advised against removing large bridges but did not specify a precise cut-off (22). Children with bars occupying less than 10%–20% of the physeal surface are considered the best candidates, given their high potential for growth recovery. Lesions involving less than 30% usually have favourable outcomes with little residual deformity. Bars affecting 30%–50% of the growth plate may still be resected if an interposition material, such as cement, fat or bone wax, is used to limit recurrence. When more than 50% of the physis is involved, outcomes are less predictable, corrective osteotomy is often necessary, and epiphysiodesis of the remaining growth plate may be considered.

Central bars generally produce limited angular deformity. In young children, treatment should therefore address the bar itself, any associated deformity and the resulting limb-length discrepancy. As illustrated by our case, physeal bar resection may restore longitudinal growth and reduce the discrepancy. For extensive bars, growth modulation of the contralateral limb can help correct residual inequality. However, late sequelae, persistent limb-length discrepancy or bar recurrence may still require additional surgery.

Several techniques have been described for removing central physeal bars. The conventional method uses a metaphyseal window or tunnel, with direct visualisation or guidance from a dental mirror. In 1981, Langenskiöld used an arthroscopy lamp, but not the arthroscope itself, to illuminate the operative field (22). In 1992, Stricker reported the first use of arthroscopic visualisation during central physeal bar resection in a single case of developmental growth arrest (23). However, this approach was limited by the difficulty of combining burring with arthroscopic viewing in a narrow, blood-filled tunnel (23). More recently, a modified arthroscopically assisted “all-inside” technique has been introduced, reducing the need for repeated fluoroscopic burring, irrigation, suction and canal arthroscopy (24).

Resection of a central physeal bar remains technically demanding, with less predictable outcomes. The main difficulties are accessing the bar without damaging the healthy peripheral growth cartilage and accurately visualising both the lesion and the surrounding normal physis. Arthroscopic assistance can improve visualisation of the peripheral physis during central bar resection and help prevent excessive removal of healthy growth cartilage. C-arm fluoroscopy also helps prevent excessive bone resection and joint penetration, particularly when epiphyseal cupping, forking or flattening is present.

Our literature review comprised 271 patients and found that almost 80% of physeal bars occurred after traumatic fractures (25). Infection was the second most frequent cause, representing 14% of cases. The distal tibia was the most affected site (42.7%), followed by the distal femur (35.4%), distal radius (10.2%) and proximal tibia (9.9%). Boys accounted for approximately 60% of cases, in line with their higher incidence of traumatic injuries. The mean age was 9.5 years (range: 2–15). Bar involvement ranged from 2.6% to 70% of the physeal surface, with a pooled weighted mean of 21.9%. Regarding location, bars were peripheral in 51.4% of cases, central in 38.6% and mixed in 10% (Table 1) (24, 25).

**Table 1:** Summary of study characteristics and pooled data from the included literature (15 studies, 271 patients). Trauma was the predominant aetiology (79.1%), followed by infection (14.2%). The most frequently affected anatomical sites were the distal tibia (42.7%) and distal femur (35.4%). Physeal bars were predominantly peripheral (51.4%), followed by central (38.6%) and mixed (10.0%) locations. The mean patient age was 9.5 years old, and the mean physeal bar coverage was 21.9% of the physeal surface.

Category	Variable	Values
Study characteristics	Number of studies	15
	Study design	Retrospective case series
	Total patients	271
Demographics	Sex (M/F)	163/108
	Male (%)	60.1%
	Female (%)	39.9%
	M:F ratio	∼1.5:1
	Mean age (years)	9.5 (range: 2.0 to 15.0)
Aetiology (reported for 210 patients)	Trauma	178/225 (79.1%)
	Infection	32/225 (14.2%)
	Other	14/225 (6.2%)
	Combined	1/225 (0.4%)
Anatomical site	Distal tibia	117 (42.7%)
	Distal femur	97 (35.4%)
	Distal radius	28 (10.2%)
	Proximal tibia	27 (9.9%)
	First metatarsal	2 (0.7%)
	Proximal femur	1 (0.4%)
	Middle finger	1 (0.4%)
Physeal bar location (reported in 210 patients)	Central	81/210 (38.6%)
	Peripheral	108/210 (51.4%)
	Mixed	21/210 (10.0%)
Bar characteristics	Mean bar coverage (%)	21.9 (range: 2.6 to 70.0)
Follow-up	Mean follow-up (months)	62 (range: 9–228)

Post-infection physeal bars predominantly affected young children, developing after infection at a mean age of 16 months and presenting at a mean age of 4.1 years. The distal femur and proximal tibia were the most involved sites (19, 20, 26, 27), and more than half of the bars (56.5%) were central. Accordingly, surgery was performed at a relatively young mean age of 6.2 years. Although exact infection-specific proportions could not be established, the reported lesions were generally large. Outcomes after resection were difficult to assess, as detailed follow-up data were available for only 15 cases. Among these, six achieved complete correction, three improved partially, and eight had poor outcomes or required additional surgery (Table 2). In this context, our case demonstrates that surgical treatment can both restore growth and improve epiphyseal morphology.

**Table 2:** Summary of the 32 post-infection physeal bar cases reported in 6 studies, including demographic characteristics, lesion location, bar size, surgical management, interposition materials, and available outcomes. Reporting of physeal bar size was heterogeneous across studies, with exact infection-specific quantitative data available only for a limited number of patients. Because only four studies provided patient-specific follow-up for infective cases, outcome analysis was limited to 15 cases and should be interpreted descriptively.

Category	Variable	Value
Study characteristics	Studies reporting post-infection bars	6
	Total number of post-infection bars	32
Sex	Male	13/22 (59.1%)
	Female	9/22 (40.9%)
Age at infection	Mean (months)	16.2
	Median and range (months)	3 (0–60)
Age at presentation	Mean (years)	4.1
	Median and range (years)	3 (0.5–10)
Age at intervention	Mean (years)	6.15
	Median and range (years)	5 (2.3–10)
Anatomical site	Distal femur	12/22 (54.5%)
	Proximal tibia	10/22 (45.5%)
Bar location	Central	13/23 (56.5%)
	Peripheral	3/23 (13.0%)
	Extensive	7/23 (30.4%)
Bar size	Mean patient-level coverage (%)	15.3 (range: 4.9 to 26.0)
	Note	Heterogeneous reporting: mixed cohorts not included
Surgical procedures	Physeal bar resection	19/32 (59.4%)
	Guided growth	8/32 (25.0%)
	Resection + guided growth	6/32 (18.8%)
	Osteotomy	7/32 (21.9%)
Interposition Materials	Materials used	Autologous fat; PMMA cement
Outcomes	Cases with detailed outcomes	15/32
	Full correction	6
	Partial improvement	3
	Further surgery required	3
	Poor outcome or recurrence	5

## 5 Conclusion

Post-infection physeal bars account for fewer than 15% of growth disturbances affecting paediatric long bone physes. They are often large, centrally located and associated with limb-length discrepancy. They may also distort epiphyseal morphology, leading to clinically relevant joint incongruity. Because infants and young children retain substantial growth potential, surgical bar resection can allow growth to resume. By restoring longitudinal growth and correcting epiphyseal deformity or irregularity, this procedure may improve both alignment and function of the affected limb segment.

## Data Availability

The original contributions presented in the study are included in the article/Supplementary Material, further inquiries can be directed to the corresponding author.

## References

[1] Dabash S Prabhakar G Potter E Thabet AM Abdelgawad A Heinrich S. Management of growth arrest: current practice and future directions. J Clin Orthop Trauma. (2018) 9:S58–66. 10.1016/j.jcot.2018.01.001 29628701 PMC5883917

[2] Wang DC Deeney V Roach JW Shah AJ. Imaging of physeal bars in children. Pediatr Radiol. (2015) 45:1403–12. 10.1007/s00247-015-3280-5 25786604

[3] Synder M Harcke H Conard K Bowen J. Experimental epiphysiodesis: magnetic resonance imaging evaluation with histopathologic correlation. Int Orthop. (2001) 25:337–42. 10.1007/s002640100271 11820437 PMC3620792

[4] Miyamura S Tanaka H Oka K Shigi A Abe S Yoshikawa H. Physeal bar resection using a patient-specific guide with intramedullary endoscopic assistance for partial physeal arrest of the distal radius. Arch Orthop Trauma Surg. (2018) 138:1179–88. 10.1007/s00402-018-2985-y 29955969 PMC6060782

[5] Ogden JA. Injury to the growth mechanisms of the immature skeleton. Skeletal Radiol. (1981) 6:237–53. 10.1007/BF00347197 7292021

[6] Peterson HA. Partial growth plate arrest and its treatment. J Pediatr Orthop. (1984) 4:246–58. 10.1097/01241398-198403000-00015 6699167

[7] Ogden JA. Skeletal Injury in the Child. 3rd edn. New York, NY: Springer-Verlag New York, Inc (2000).

[8] Peterson HA. Epiphyseal Growth Plate Fractures. Berlin, Heidelberg: Springer Berlin Heidelberg (2007). p. 21–91 10.1007/978-3-540-33802-4_3

[9] Singh V Garg V Parikh SN. Management of physeal fractures: a review article. Indian J Orthop. (2021) 55:525–38. 10.1007/s43465-020-00338-6 33995857 PMC8081798

[10] Jawetz ST Shah PH Potter HG. Imaging of physeal injury: overuse. Sports Health. (2015) 7:142–53. 10.1177/1941738114559380 25984260 PMC4332644

[11] Lohman M Kivisaari A Vehmas T Kallio P Puntila J Kivisaari L. MRI In the assessment of growth arrest. Pediatr Radiol. (2002) 32:41–5. 10.1007/s00247-001-0572-8 11819062

[12] Borsa JJ Peterson HA Ehman RL. MR Imaging of physeal bars. Radiology. (1996) 199:683–7. 10.1148/radiology.199.3.8637987 8637987

[13] Sailhan F Chotel F Guibal A-L Gollogly S Adam P Bérard J. Three-dimensional MR imaging in the assessment of physeal growth arrest. Eur Radiol. (2004) 14:1600–8. 10.1007/s00330-004-2319-z 15064854

[14] Pai M McCulloch M Gorman JD Pai N Enanoria W Kennedy G. Systematic reviews and meta-analyses: an illustrated, step-by-step guide. Natl Med J India. (2004) 17:86–95. 15141602

[15] Peterson HA. Physeal Injury Other Than Fracture. Berlin, Heidelberg: Springer Berlin Heidelberg (2012). 10.1007/978-3-642-22563-5

[16] Agarwal A Jethwa R. Post infective physeal bar sequelae around knee: natural history and coronal plane deformities. J Clin Orthop Trauma. (2023) 41:102176. 10.1016/j.jcot.2023.102176 37483914 PMC10362537

[17] Hernandez J Peterson HA. Fracture of the distal radial physis complicated by compartment syndrome and premature physeal closure. J Pediatr Orthop. (1986) 6:627–30. 10.1097/01241398-198609000-00020 3760178

[18] Agarwal A Aggarwal AN. Bone and joint infections in children: acute hematogenous osteomyelitis. Indian J Pediatr. (2016) 83:817–24. 10.1007/s12098-015-1806-3 26096866

[19] Masquijo JJ Allende V Ferreyra A Hernández Bueno JC. Distal femoral physeal bar resection combined with guided growth for the treatment of angular limb deformity associated with growth arrest: a preliminary report. J Pediatr Orthop. (2020) 40:e958–62. 10.1097/BPO.0000000000001651 32773655

[20] Yuan BJ Stans AA Larson DR Peterson HA. Excision of physeal bars of the distal femur, proximal and distal tibia followed to maturity. Journal of Pediatric Orthopaedics. (2019) 39:e422–9. 10.1097/BPO.0000000000001349 30817419

[21] Khoshhal KI Kiefer GN. Physeal bridge resection. J Am Acad Orthop Sur. (2005) 13:47–58. 10.5435/00124635-200501000-00007 15712982

[22] Langenskiöld A. Surgical treatment of partial closure of the growth plate. J Pediatr Orthop. (1981) 1:3–11. 10.1097/01241398-198101010-00002 7341649

[23] Stricker S. Arthroscopic visualization during excision of a central physeal bar. J Pediatr Orthop. (1992) 12:544–6. 10.1097/01241398-199207000-00026 1613105

[24] Xiao H Li M Tan Q Ye W Wu J Mei H. Physeal bar resection by modified arthroscopically assisted surgery in a closed osteocavity. Front Pediatr. (2023) 11:1157192. 10.3389/fped.2023.1157192 37915984 PMC10616236

[25] Cass JR Peterson HA. Salter-Harris type-IV injuries of the distal tibial epiphyseal growth plate, with emphasis on those involving the medial malleolus. J Bone Joint Surg Am. (1983) 65:1059–70. 10.2106/00004623-198365080-00005 6630250

[26] Marsh JS Polzhofer GK. Arthroscopically assisted central physeal bar resection. J Pediatr Orthop. (2006) 26:255–9. 10.1097/01.bpo.0000218533.43986.e1 16557145

[27] Hasler CC Foster BK. Secondary tethers after physeal bar resection: a common source of failure? Clin Orthop Relat Res. (2002) 405:242–9. 10.1097/00003086-200212000-00031 12461380

